# Microplastics Affect the Inflammation Pathway in Human Gingival Fibroblasts: A Study in the Adriatic Sea

**DOI:** 10.3390/ijerph19137782

**Published:** 2022-06-24

**Authors:** Sergio Caputi, Francesca Diomede, Paola Lanuti, Guya Diletta Marconi, Piero Di Carlo, Bruna Sinjari, Oriana Trubiani

**Affiliations:** 1 Department of Innovative Technologies in Medicine & Dentistry, University “G. d’Annunzio” Chieti-Pescara, Via dei Vestini, 31, 66100 Chieti, Italy; sergio.caputi@unich.it (S.C.); francesca.diomede@unich.it (F.D.); piero.dicarlo@unich.it (P.D.C.); 2 Department of Medicine and Aging Sciences, University “G. d’Annunzio” Chieti-Pescara, Via dei Vestini, 31, 66100 Chieti, Italy; paola.lanuti@unich.it; 3 Department of Medical, Oral and Biotechnological Sciences, University “G. d’Annunzio” Chieti-Pescara, Via dei Vestini, 31, 66100 Chieti, Italy; guya.marconi@unich.it

**Keywords:** sea, microplastics, pollution, human gingival fibroblasts, intracellular inflammation pathway

## Abstract

The level of environmental microplastics in the sea is constantly increasing. They can enter the human body with food, be absorbed through the gut and have negative effects on the organism’s health after its digestion. To date, microplastics (MPs) are considered new environmental pollutants in the air sea and they are attracting wide attention. The possible toxic effects of MPs isolated at different sea depths of 1, 24 and 78 m were explored in an in vitro model of human gingival fibroblasts (hGFs). MPs isolated from the sea showed different size and were then divided into different sample groups: 1, 24 and 78 m. The results obtained revealed that MPs are able to activate the inflammatory pathway NFkB/MyD88/NLRP3. In detail, the exposure to MPs from 1 and 78 m led to increased levels of inflammatory markers NFkB, MyD88 and NLRP3 in terms of proteins and gene expression. Moreover, cells exposed to MPs showed a lower metabolic activity rate compared to unexposed cells. In conclusion, these findings demonstrate that the inflammation process is stimulated by MPs exposure, providing a new perspective to better understand the intracellular mechanism.

## 1. Introduction

The continuous consumption of plastic by modern society leads to a consequent increase in the production of plastic waste [1]. This causes a slowdown in waste management and recycling processes, which leads to an accumulation of plastic in environments including the marine one [2]. It was estimated that plastic fragments represented 60%–80% of total marine debris and >90% of floating particles making them the predominant components of marine debris. This situation has alerted both the scientific community and policy makers. In 2014, the United Nations Environment Program (UNEP) identified plastic pollution in the oceans as one of the top 10 emerging global environmental problems [3]. Plastic pollution has several negative effects in the marine environment: alterations in biodiversity and ecosystem health, entanglement and ingestion by marine biota, leaching of chemicals, and socio-economic consequences related to tourism, navigation, fishing and aquaculture activities [4].

In general, plastic is very resistant to decomposition and, for this reason, it can remain in the environment and pollute it for centuries. Most plastics are resistant to biodegradation, but they will break down gradually through mechanical action. Plastic waste decomposes into small plastic fragments of 5–40 mm, then into microplastics (MPs, plastic particles with dimensions of 1–5000 μm) and nanoplastics (NPs), particles unintentionally produced within the size range of 1 to 1000 nm [5,6].

Specifically, MPs are distinguished into primary and secondary according to the sources from which they derive: primary MPs are those released directly into the environment in the form of micropellets, microspheres or microfibers, while secondary MPs are those derived from the rupture of large or meso-litter plastics in the environment under the action of physical, chemical and biological forces. Most of the MPs found in the environment are secondary MPs [7,8]. There are many kinds of MPs. Polystyrene (PS) was found to be one of the main components of MPs pollution in the environment and it is widely used in the production of plastic products and plastic packaging [9]. Due to the increase in consumption of MPs, their potential effects in human health have become the focus of researchers [10]. Studies have shown that food intake is one of the main pathways for MPs to enter the organism and accumulate in tissues and organs [11,12]. Animal studies have reported intestinal inflammation, intestinal villi rupture, intestinal epithelial cell damage and intestinal metabolism disorder after exposure to MPs [13]. The toxicity of MPs is size-dependent [14]. Since MPs particles mostly enter the human body together with food and the digestive tract is the main place for digestion and absorption of food, it is important to understand the impact of MPs in human health [15]. The oral cavity is the first part of the gastro-intestinal apparatus and it is the first point of contact with MPs in food. 

Human gingival fibroblasts extracted from masticatory gingival tissue showed unique morphological features such as a spindle-shaped appearance and adherent growth on plastic [16,17]; moreover, human gingival fibroblasts (hGFs) play a crucial role in the secretion and degradation of the extracellular collagen matrix, which provides structural integrity and participates in wound healing and tissue regeneration, immunological regulation, mechano-transduction, angiogenesis and organ fibrosis [18,19]. MPs could also be a risk factor for the development of Autism Spectrum Disorder, and the maternal ingestion of MPs could represent a risk factor for the development of ASD [20].

Several scientific data reported the presence of both plastics and MPs in populated coastal areas and in remote parts of the world such as polar seas and deep abyssal environments [4].

The Mediterranean Sea was one of the different seas considered in the studies for analysis of microplastic pollution. Large amounts of plastic debris have been reported from the Mediterranean seabed and floating on its surface, as well as on beaches and coastal environments. Mediterranean biodiversity is affected by the interaction with marine litter. Artificial polymers have been found in the stomach contents of Mediterranean pelagic predators, deep-sea fish and commercial species [21]. About 1/3 of Mediterranean freshwater discharges flow into the Adriatic Sea, which is a narrow elongated sub-basin with a high land to sea ratio (1.80) surrounded by seven countries (~3.5 million inhabitants in the coastal zone) and many tourist centers (e.g., Venice, Split, Dubrovnik, Corfu) [22,23]. Our study was focused mainly on the toxicity of the MPs identified in the Adriatic Sea to assess the risk they pose to human health. In the current work, water samples withdrawn from three different depths of the Adriatic Sea have been studied: 1, 24, and 78 m. In these samples, MPs of different sizes were found in the water taken at a depth of 1 m, MPs in the range of 100–150 nm were found in the water at 1 and 78 m, while in the water at 24 m depth, MPs were found in the range of 600–700 nm. In the present study, the effect of MPs of different sizes and at different depths was evaluated on cellular toxicity using human gingival fibroblasts (hGFs) in order to evaluate the role of sea MPs on inflammation by analyzing inflammation pathways. 

## 2. Materials and Methods

### 2.1. Sampling Procedures and Estimation of Microplastics (MPs) Recovery

**Figure 1:**
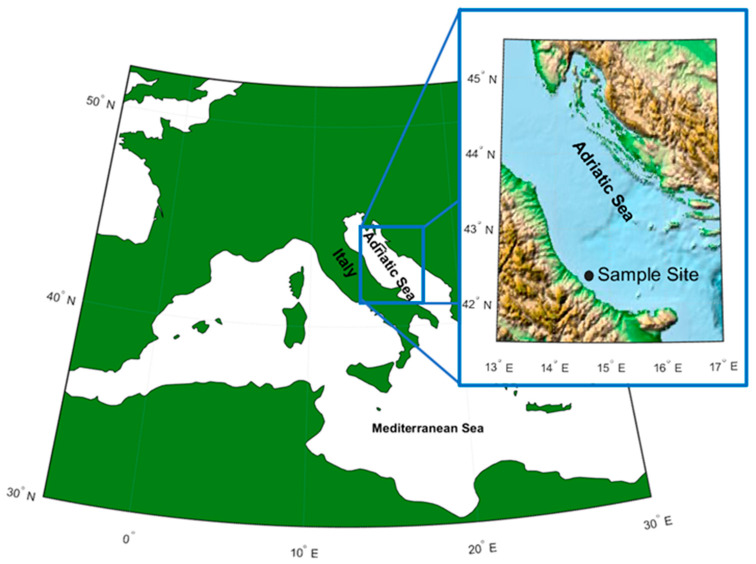
Map of the Adriatic Sea where the pilot study was carried out.

The Adriatic Sea is a part of the Mediterranean basin, extending from the Italian Peninsula to the west and the Balkan Peninsula to the east. It is about 800 km long and 90 to 220 km wide, and extends between 40° and 46° lat. N and between 12° and 20° across (Figure 1). The area of the Adriatic Sea is about 132,000 km^2^, the average depth ranges from about 35 (the northern part) to 140 m (central part), reaching 260 m in the Pomo Depressions. The Otranto Canal is the connection between the Adriatic Sea and Mediterranean Sea. 

The water dynamic of the Adriatic Sea is mainly cyclonic, driven by thermohaline currents with rare storms that can result in waves exceeding 9 m in amplitude [24]. The water mass of the Adriatic Sea is well stratified and can be divided in three main layers: (1) a superficial layer (0–30 m) with the upper 10 m being less saline, dominated by waters of coastal origin; (2) a Levantine intermediate water layer (30–130 m) with maximum salinity at about 80 m depth; and (3) a bottom-water region (>130 m) with very dense waters [25]. 

The sea water specimens analyzed in this work were sampled from the central Adriatic (Geographical fishing Sub-Area, GSA17) during a pilot study carried out in summer–fall 2019 focused on the collection of marine litter with the help of 40 fishing boats. The collected marine litters were intercepted by trawls and deposited in the Pescara harbor in central Italy for litter classification and evaluation. Besides marine litter analysis, sea water specimens were collected from three different depths from the sea surface (1, 24 and 78 m) to have samples of the different layers of the Adriatic and to check if the composition and presence of micro and nano-plastics is depth-dependent. The site of the sample (Figure 1) was 13 km from the coast (42°26′64″ N, 14°31′63″ E).

### 2.2. Cell Culture Establishment

Primary cultures of human gingival fibroblasts (hGFs) were established by the explant method as previously described [26]. Fragments of healthy gingival tissue were rinsed three times in Phosphate Buffered Saline (PBS, Lonza, Basel, Switzerland) solution, cut into small tissue pieces and cultured in Dulbecco’s modified Eagle’s medium (DMEM, Lonza) supplemented with 10% Fetal Bovine Serum (FBS, Lonza) and 0.1% gentamicin (10 mg/mL; Euroclone, Milan, Italy) at 37 °C in 5% CO_2_ atmosphere. The gingival tissue biopsies were cultured until hGFs spontaneously migrated (about 4 weeks; [27]). Cells were incubated in standard conditions (37 °C in a humidified atmosphere of 5% (*v*/*v*) CO_2_). Cells were observed under inverted light microscopy (Leica Microsystem, Milan, Italy) as previously described [28]. All the experiments were performed with cells processed between 4 and 8 passages and each assay was performed in triplicate.

### 2.3. Flow Cytometry Analyses of Particle Size

Water samples collected at 1, 24 and 78 m were treated with 1% Triton X-100 (30 min) in order to remove any biological particles. Samples were then acquired by flow cytometry (FASCVerse, BD Biosciences, San Jose, CA, USA). To avoid swarm effects, sample dilution was established as recommended [29]. In any case, the flow rate resulted in event counts ≤2000 events/second. Diluted samples were then acquired and 1 × 10^5^ events/sample were registered. Side Scatter and Forward Scatter (FSC) MegaMix-Plus beads (Byocitex, Marseille, France) were used to measure the particle size [30,31]. MegaMix-Plus SSC and FSC are mixes of fluorescent polystyrene beads of known diameters selected to cover the size range 0.1–1 μm (SSC beads: 0.16, 0.20, 0.24 and 0.50 μm; FSC beads: 0.1, 0.3, 0.5 and 0.9 μm). By acquiring the beads using the same setting applied for sample analysis, it is possible to identify, in the scatter plots, the particle diameter ranges, given that the scattered light (FSC and/or SSC) is proportional to the particle diameter [32]. The same parameters were applied for all other analyses. Instrument performances, data reproducibility and fluorescence calibrations were sustained by the Cytometer Setup & Tracking Module (BD Biosciences) [32]. 

### 2.4. Study Design

All the following experiments were performed in triplicate. The present work was carried out with the following groups:

– Untreated hGFs, used as control (CTRL);
– hGFs treated with 100 nm micro plastics isolated at 1 m depth (1 m);
– hGFs treated with 600 nm micro plastics isolated at 24 m depth (24 m).
– hGFs treated with 100 nm micro plastics isolated at 78 m depth (78 m);

### 2.5. Cell Metabolic Activity

hGFs were seeded at a cell density of 2 × 10^3^ cells/well into a 96-well tissue culture plate. The cell metabolic activity of hGFs was evaluated after 24, 48 and 72 h of treatment in all sample groups: 1, 24 and 78 m. The 3-(4,5-dimethylthiazol-2-yl)-5-(3-carboxymethoxyphenyl)-2-(4-sulfo-phenyl)-2H-tetrazolium (MTS) assay (CellTiter 96^®^ Aqueous One Solution Cell Proliferation Assay, Promega, Madison, WI, USA) was used. At the established end points, 20 μL/well of MTS dye solution was added to culture medium, and cells were incubated for 3 h at 37 °C. The quantity of formazan product, directly proportional to the number of living cells in culture, was detected by absorbance measurements at 490 nm wavelength utilizing the Synergy™ HT Multi-detection microplate reader (Biotech, Winooski, VT, USA). The MTS assay was executed in three independent experiments.

### 2.6. Immunofluorescence Analyses

All samples were processed for observation under confocal laser scanning microscopy (CLSM). Cells were exposed to the MPs (derived from 1, 24 and 78 m) for 48 h of culture. A 4% solution of para-formaldehyde in 0.1 M PBS (Lonza) was used to fix cells. The permeabilization process was then performed by means of treatment with 0.5% Triton X-100 in PBS (Lonza) for 10 min followed by blocking with 5% skimmed milk in PBS (Lonza) for 30 min. Subsequently, the cells were incubated for 2 h at room temperature with the following primary antibodies: anti-NF-kB (1:500; SantaCruz Biotechnology, Dallas, TX, USA), anti-MyD88 (1:500, SantaCruz Biotechnology) and anti NLRP3 (3 µg/mL; Novus, Milan, Italy). Alexa Fluor 568 red fluorescence conjugate was used as secondary antibody (1:200; Molecular Probes, Thermo Fisher Scientific), samples were incubated for 1 h at 37 °C [33]. Cells were then stained for 1 h with Alexa Fluor 488 phalloidin green fluorescent conjugate (1:400; Molecular Probes) to mark cytoskeleton actin and for 1 h with TOPRO (1:200; Molecular Probes) to stain cell nuclei. Glass coverslips were placed upside down on glass slides and mounted with Pro-Long Gold Antifade (Molecular Probes). CLSM (LSM800, Zeiss, Jena, Germany) was used to visualize the samples. All images were acquired at a resolution of 1024 × 1024 pixels at 12 bit (4096 grey values) using ZEN 3.0 SR software (Zeiss).

### 2.7. RNA Isolation and Real-Time PCR Analysis

NFkB, MyD88 and NLRP3 mRNAs expression was analyzed by real-time PCR on hGFs exposed to the MPs (1, 24 and 78 m) for 48 h. Total RNA was extracted using a PureLink RNA Mini Kit (Ambion, Thermo Fisher Scientific, Milan, Italy) according to the manufacturer’s instructions. Three independent biological replicates were analyzed for each sample. RNA (2 μg) was retrotranscribed using the High Capacity cDNA Reverse Transcription Kit catalog number 4,368,814 (Applied Biosystems, Waltham, MA, USA) to synthesize cDNA for 10 min at 25 °C, 10 min at 37 °C and 5 min at 85 °C according to the manufacturer’s instructions. Real-time PCR was performed with the Mastercycler ep real plex real-time PCR system (Eppendorf, Hamburg, Germany). The levels of mRNA expression of NFkB, MyD88, NLRP3 and Beta-2 microglobulin (B2M) (endogenous marker) were evaluated in hGFs cells exposed to MPs from 1, 24 and 78 m depth compared to the mRNA expression levels of unexposed hGFs (CTRL). Commercially available TaqMan Gene Expression Assays (NFkB Hs.PT.58.22880470; MyD88 Hs.PT.58.40428647.g; NLRP3 Hs.PT.58.39303321; TemaRicerca, Milan, Italy) and the TaqMan Universal PCR Master Mix (Applied Biosystems, Foster City, CA, USA) were utilized according to standard protocols. Beta-2 microglobulin (B2M Hs99999907_m1) was utilized for template normalization. The amplification program consisted of a pre-incubation step for cDNA denaturation (3 min 95 °C), followed by 40 cycles consisting of a denaturation step (15 s 95 °C) and an annealing step (1 min 60 °C) [34]. At the end of each run, melting curve analysis was performed in the temperature range of 60 °C to 95 °C. Expression levels for each gene were performed according to the 2^−ΔΔCT^ method. RT-PCR was performed in three independent experiments; duplicate determinations were performed for each specimen.

### 2.8. Western Blot Analysis

Proteins from untreated and MPs-treated hGFs (for 48 h) were separated using sodium dodecyl-sulfate polyacrylamide gel electrophoresis (SDS-PAGE) followed by Western blot analysis (Bio-Rad V3 Western Workflow™, Milan, Italy). Membranes were saturated for 120 min at room temperature in a blocking buffer (1 × TBS, 5% milk, 0.1% Tween-20) followed by overnight incubation at 4 °C with the following primary antibodies: mouse anti-NFkB (1:500; Santa Cruz Biotechnology), mouse anti-MyD88 (1:500; Santa Cruz Biotechnology) and mouse anti-NLRP3 (3 µg/mL; Novus). Subsequently, membranes were incubated for 60 min at room temperature with peroxidase-conjugated anti-mouse secondary antibody (1:5000; Bethyl Laboratories, Montgomery, AL, USA) [35]. Enhanced chemiluminescence with the Alliance 2.7 system (Uvitec Ltd., Cambridge, UK) was used to identify and quantify the bands obtained.

### 2.9. Statistical Analysis

GraphPad Prism software (version 5.01, GraphPad Software, San Diego, CA, USA) was used for the statistical analysis. The data were analyzed using one-way ANOVA followed by Tukey’s post hoc test for a comparison of the means of all groups with the mean of all other groups. *p* < 0.05 was considered statistically significant. Data were expressed as the mean ± S.E.M. All data were collected for at least three independent experiments.

## 3. Results

### 3.1. MPs Characterization

**Figure 2:**
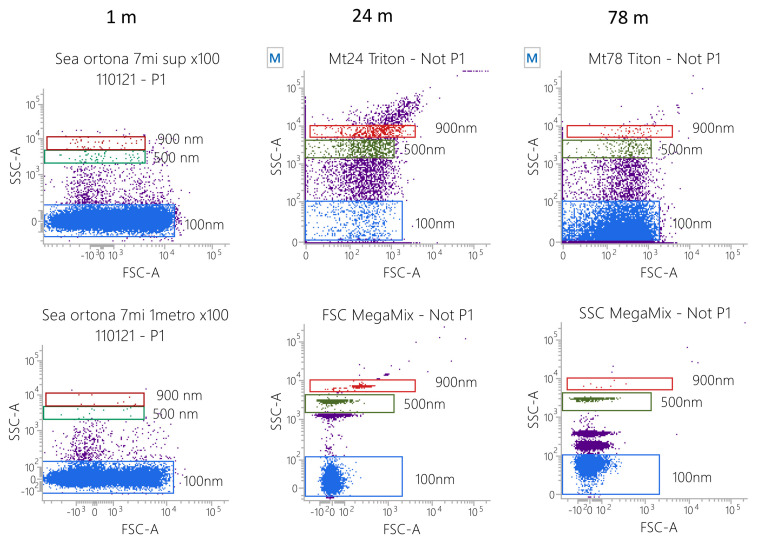
Flow cytometry analysis of particle features. FSC MegaMix-Plus and SSC MegaMix-Plus were used as a reference to identify size ranges on a FSC-A/SSC-A dot plot. Water samples at different depths were analyzed using the same parameters applied for bead acquisition. In detail, the following samples were collected and analyzed: water surface, 1 m depth, 24 m depth and 78 m depth.

The inorganic particles in the water samples taken at 1, 24 and 78 m were acquired by flow cytometry using an appropriate setting [25]. MegaMix-Plus beads (beads with known diameters) were used as reference materials to set the gates, identifying specific diameter values (low dot plots; 100, 500 and 900 nm). When samples were taken at low sea depths (1–7 m) the smallest particles (about 100 nm in diameter), which are probably the lighter ones, were detected. At middle depths (24 m) bigger particles (100–1000 nm) were observed, while at higher sea depths (78 m) a mix of small (about 100 nm, the densest) and medium particles (200–500 nm) was identified (Figure 2).

### 3.2. MPs Attenuated the Cell Viability Rate of hGFs

**Figure 3:**
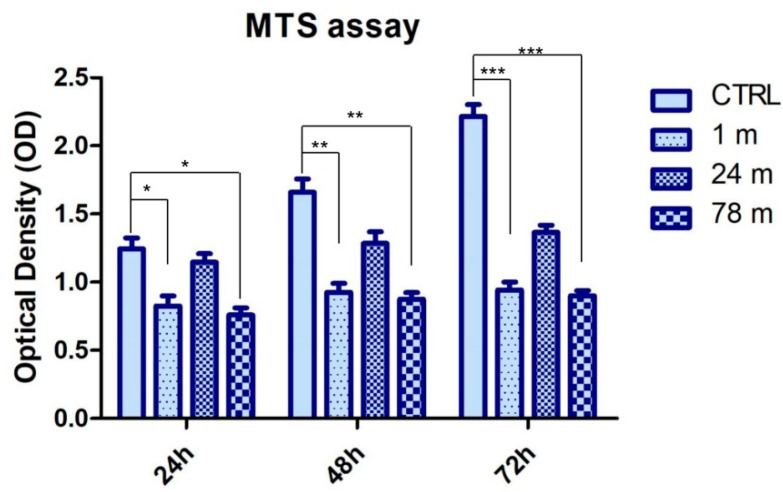
Graph bars indicating the optical density obtained by means of MTS assay at 24, 48 and 72 h of culture. * *p* < 0.05; ** *p* < 0.01; *** *p* < 0.001.

Bar graph data shows that cell viability was decreased in hGFs exposed to 1 and 78 m MPs in all considered endpoints, while the cell viability of the hGFs treated with 24 m MPs was better (Figure 3).

### 3.3. Upregulation of NFkB, MyD88 and NLRP3 Expression in hGFs Treated with MPs

**Figure 4:**
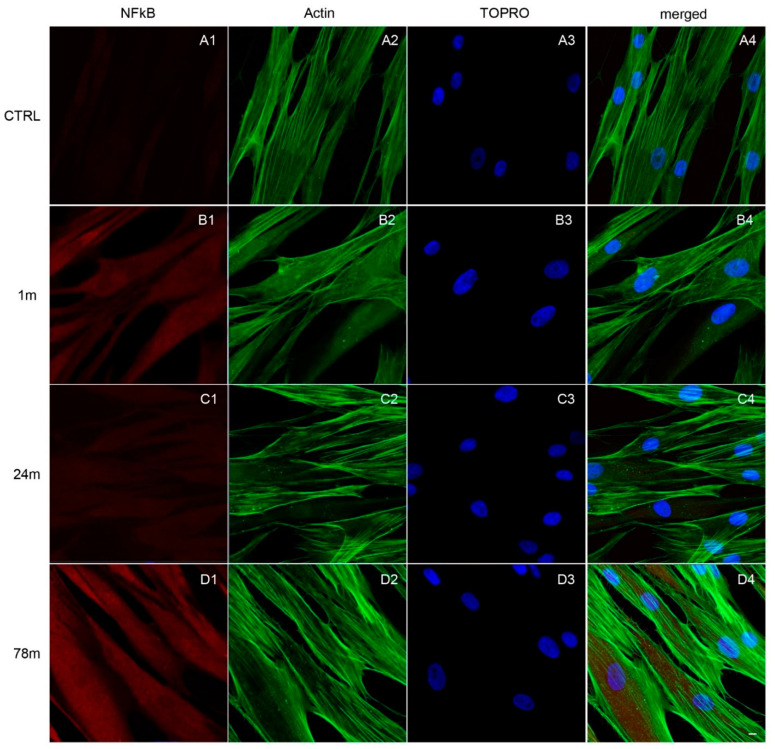
Immunofluorescence analysis of NFkB expression on (**A1**) CTRL cells and on cells exposed to (**B1**) 1, (**C1**) 24 and (**D1**) 78 m MPs. (**A1**–**D1**) Red fluorescence: cytoskeleton actin; (**A2**–**D2**) green fluorescence: NFkB; (**A3**–**D3**) blue fluorescence: cell nuclei; (**A4**–**D4**) merged image of the above-mentioned channels. Scale bar: 20 µm.

**Figure 5:**
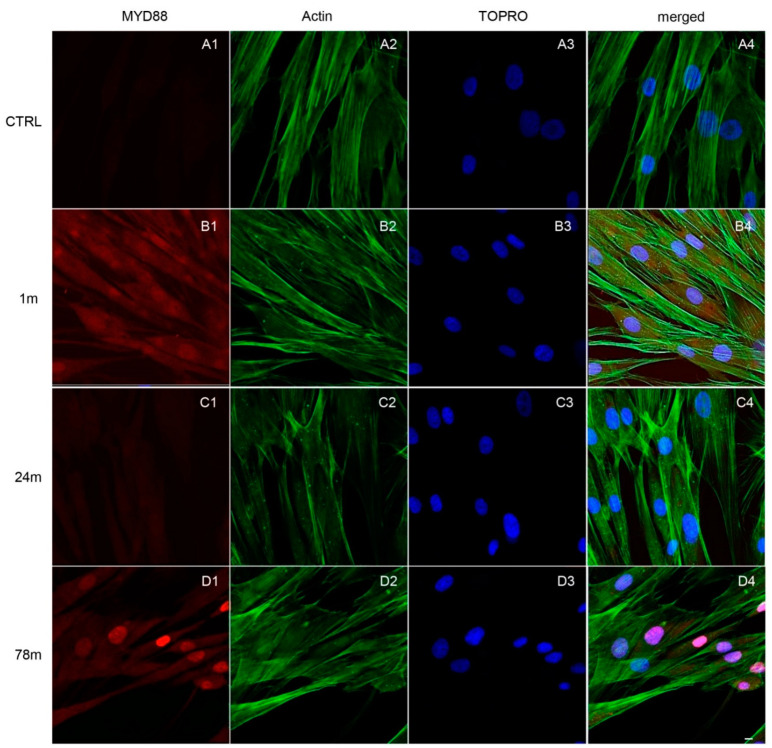
Immunofluorescence analysis of MYD88 expression on (**A1**) CTRL cells and on cells exposed to (**B1**) 1, (**C1**) 24 and (**D1**) 78 m MPs. (**A1**–**D1**) Red fluorescence: cytoskeleton actin; (**A2**–**D2**) green fluorescence: MYD88; (**A3**–**D3**) blue fluorescence: cell nuclei; (**A4**–**D4**) merged image of the above-mentioned channels. Scale bar: 20 µm.

**Figure 6:**
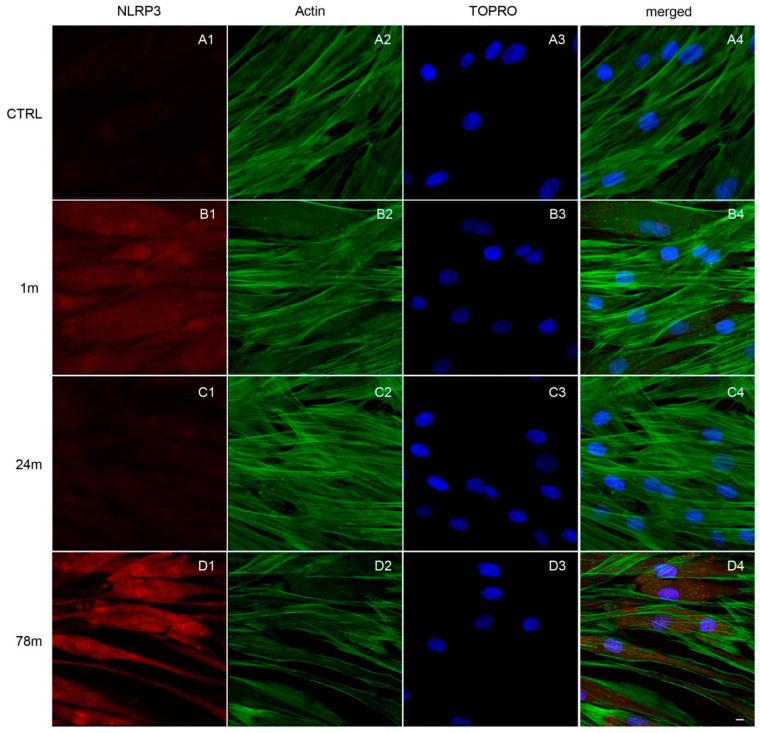
Immunofluorescence analysis of NLRP3 expression on (**A1**) CTRL cells and on cells exposed to (**B1**) 1, (**C1**) 24 and (**D1**) 78 m MPs. (**A1**–**D1**) Red fluorescence: NLRP3; (**A2**–**D2**) green fluorescence: cytoskeleton actin; (**A3**–**D3**) blue fluorescence: cell nuclei; (**A4**–**D4**) merged image of the above mentioned channels. Scale bar: 20 µm.

**Figure 7:**
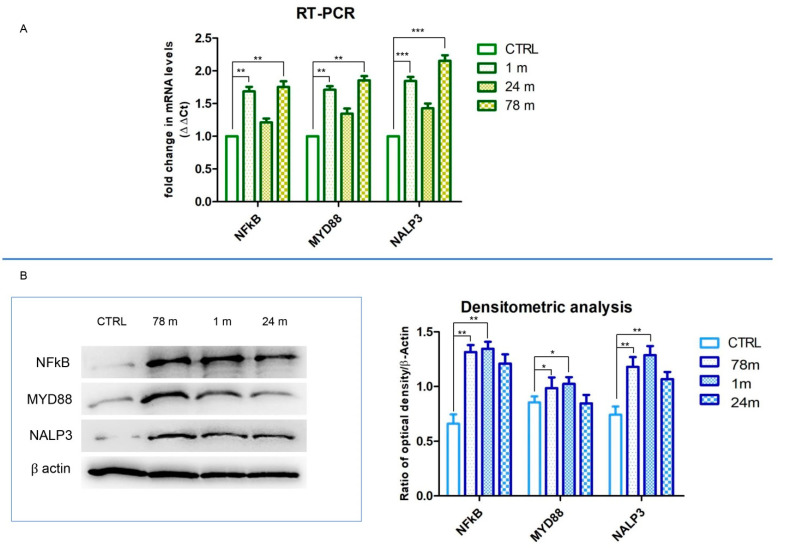
(**A**) Gene expression of NFkB, MyD88 and NLRP3. (**B**) Western blot analyses of NFkB, MyD88 and NLRP3 and relative densitometric analyses. * *p* < 0.05; ** *p* < 0.01; *** *p* < 0.001.

Immunofluorescence detection showed the expression of the inflammation pathway modulated by the exposure to MPs. As shown in Figure 4, Figure 5 and Figure 6, MPs resulted in increased expression of inflammation markers, suggesting that MPs might cause alterations in hGFs. CTRL cells showed a regular shape and were neatly arranged on a plastic substrate with no positive signals of inflammatory markers (Figure 4(A1), Figure 5(A1) and Figure 6(A1)). However, inflammatory reaction was observed in hGFs exposed to MPs (1 m and 78 m) (Figure 5(B1,D1), Figure 6(B1,D1) and Figure 7(B1,D1)). NFkB, MyD88 and NLRP3 were upregulated in cells treated with 1 and 78 m MPs, while a lesser extent was observed in cells exposed to 24 m MPs (Figure 4(C1), Figure 5(C1) and Figure 6(C1)).

To explore the specific inflammatory pathway modulation induced by MPs, the expression of NFkB, MyD88 and NLRP3 were detected by the evaluation of RT-PCR and Western blot analyses (Figure 7A,B). RT-PCR results showed that the mRNA levels of NFkB, MyD88 and NLRP3 were upregulated in cells exposed to 1 and 78 m MPs when compared to the CTRL group (unexposed cells) (Figure 7A). On the other hand, hGFs exposed to the 24 m MPs showed a slight increase in mRNA levels of all considered inflammatory markers (Figure 7A). 

Western blotting analysis showed an upregulation of NFkB, MyD88 and NLRP3 protein levels in hGFs exposed to the MPs as demonstrated by specific expression of protein bands and the related densitometric analysis. Human GFs exposed to 24 m MPs showed a slow increase in inflammatory protein expression compared to the other MPs groups (1 and 78 m) (Figure 7B).

## 4. Discussion

In the sea, the presence of more plastic products broken down into small pieces provokes the deterioration of the marine environment. The tiny pieces of plastic particles showed a diameter less than 5 mm and they can still be ingested by animals and humans [5,36,37,38,39].

Increased accumulation of MPs decomposed from plastic waste leads to a contamination in each cubic meter of ocean water [40], and they are ubiquitously distributed in open seas and coasts [41]. The potential negative effects on human health have become an important field of research [10].

MPs with different sizes could be easily absorbed by marine fauna and through the food chain; the MPs could be ingested by human organisms leading to negative effects on the human gut [42,43,44]. As reported in the recent literature, MPs can also cause harm to the respiratory tract and reproductive system of animals [45,46,47].

Some studies have summarized the possible mechanisms that contribute to negative effects on human health as cell internalization of MPs particles producing cytotoxic effects [42,48,49], increased levels of intracellular reactive oxygen species (ROS), the induction of DNA damage and the activation of pro-inflammatory cytokine release [50].

Since cells of oral cavity mucosa are in direct contact with the external environment, we speculated that the increase in MPs pollution might affect cellular physiological processes. The purpose of this study was to evaluate the effects of MPs on hGFs by using MPs derived from different sea depths and of different sizes (for sea depth: 1, 24 and 78 m; for MPs size: 100 and 600 nm) with an exposure lasting 48 h. MPs showed different degrees of cytotoxic and damaging effects on human cell lines [51,52,53]. Recent in vitro studies demonstrated the cytotoxic effects of MPs on human monocytes [54], an increase in the production of ROS in T98G and HeLa cells [55] and mitochondrial depolarization in colon adenocarcinoma cells [15]. 

The cytotoxicity of MPs was evaluated through cell metabolic activity rate experiments. Our data demonstrated that cells exposed to MPs from 1 and 78 m showed a lower cell metabolic activity rate when compared to the sample group exposed to MPs from 24 m. This process could be related to the increase in cellular oxidative stress, ROS accumulation and mitochondrial depolarization. MPs are able to enter into cells at the cytoplasmic level and increase cell toxicity [56]. Recent studies have reported that MPs accumulate in the body are able to induce oxidative stress in the gut. Wu et al. demonstrated that when Caco-2 cells were exposed to MPs in the high-concentration group, the levels of relevant antioxidant enzymes were significantly reduced, indicating that a high concentration of MPs would exert a certain inhibition on antioxidant cellular mechanisms in relation to the size of MPs [15]. Our results demonstrated that the viability rate of cells was lower when MPs were 600 nm in size, which further proved a toxic effect on hGFs when compared to the 100 nm MPs derived from 1 and 78 m of sea depth.

The lower metabolic activity could indicate that the properties of the used MPs interfere with the viability of the cells. The cytotoxicity of MPs was broadly in line with previous research [57,58]. 

The recent literature indicates that environmental pollutants, such as MPs, could trigger NLRP3 inflammasome [59,60] and increase the inflammatory factors IL-6, IL-1β and TNF-α [61,62]. Our results demonstrated that exposure to MPs leads to an increased expression of NFkB, MyD88 and NLRP3, suggesting that MPs could contribute to inducing the intracellular inflammation process [63]. NLRP3 inflammasome is the active center for regulation of the cellular inflammatory response [64,65,66]. 

Interestingly, in our experiments, we observed a significant increase in NFkB, MyD88 and NLRP3 expression after exposure to MPs from 1 and 78 m, while the expression of the investigated inflammatory markers was downregulated when cells were exposed to MPs from 24 m, as reported in the immunofluorescence results and validated by Western blotting analysis.

The protein molecular expression and gene expression trends in this study are consistent with the qualitative data obtained by immunofluorescence experiments. Oxidative stress and pro-inflammatory processes were considered to be interdependent, but many studies have shown that oxidative stress can induce inflammatory responses and the release of many chemokines [64,67]. Environmental toxicants showed deleterious effects on female reproduction in utero, in the neonatal and prepubertal periods and in adulthood [68]. Toxicant exposure during the development of the tooth germ leads to the occurrence of structural anomalies in teeth [69].

NFkB plays a key role in the cellular response to oxidative stress and is also largely considered as an activator of classic pro-inflammatory signaling pathways [70,71]. As previously demonstrated, MPs are able to induce a strong cellular inflammatory response [72]. NLRP3 inflammasome activation is strictly related to NFkB expression, and the inhibition of NFkB reduces the pro-inflammatory response mediated by the NLRP3 inflammasome. MyD88 is a molecular factor that modulates most TLR signaling, as well as Interleukin (IL)-1, IL-18 receptors and Toll-like receptors (TLRs) activating the innate immune response and the inflammation process [73]. The innate immune response during inflammation is regulated by the TLRs via the expression of NFkB that leads to an increasing release of inflammatory cytokines and chemokines [74]. Obtained results demonstrated an increasing level of inflammatory related markers in hGFs exposed to MPs from 1 and 78 m, and this activation could be correlated to the size of MPs (100 nm).

## 5. Conclusions

Our results provided a novel insight on MPs-induced inflammation and illustrated that exposure to MPs induces the inflammation process in hGFs via the activation of the NFkB/MyD88/NLRP3 pathway. In conclusion, MPs obtained from 1 and 78 m sea depth can trigger inflammatory responses in human oral cells.

## Data Availability

Data are available at the corresponding authors upon request.
